# A Critical Overview of Systematic Reviews of Shenfu Injection for Heart Failure

**DOI:** 10.1155/2021/8816590

**Published:** 2021-03-06

**Authors:** Jinke Huang, Yanlu Wang, Suihe Huang, Xiaohui Qin, Fang Yan, Min Shen, Yong Huang

**Affiliations:** ^1^The Second Clinical Medical College of Guangzhou University of Chinese Medicine, Guangzhou, Guangdong Province 510120, China; ^2^The First Clinical Medical College of Shandong University of Traditional Chinese Medicine, Jinan, Shandong Province 255014, China; ^3^Research Base of the Clinical Application of Traditional Chinese Medicine Classics, Guangdong Provincial Hospital of Chinese Medicine, Guangzhou, Guangdong Province 510120, China; ^4^Department of Neurology, Guangdong Provincial Hospital of Chinese Medicine, Guangzhou, Guangdong Province 510120, China; ^5^School of Traditional Chinese Medicine, Southern Medical University, Guangzhou, Guangdong Province 510515, China

## Abstract

**Objectives:**

Shenfu Injection (SFI) was widely used in the treatment of heart failure (HF) in China. A plethora of systematic reviews/meta-analyses (SRs/MAs) has been conducted in this research area, although with scattered results. The purpose of this overview was to conduct a comprehensive review to summarize and critically evaluate the existing evidence.

**Methods:**

Digital databases were searched for SRs/MAs up to January 28, 2021. Two authors independently screened the reviews and assessed the methodological quality of included SRs/MAs using Assessing the Methodological Quality of Systematic Reviews 2 (AMSTAR-2). Quality of evidence for outcomes evaluated within the reviews was appraised with the Grading of Recommendation, Assessment, Development, and Evaluation (GRADE).

**Results:**

Thirteen SRs/MAs met the inclusion criteria. Based on AMSTAR-2, the quality of all SRs/MAs was critically low, because all of them have more than one critical domains that were unmet. Based on GRADE, the evidence quality of 24 outcome measures was low or very low, 27 outcome measures was moderate, and none outcome measure was high. Descriptive analysis showed that SFI was an effective and safe method for HF.

**Conclusions:**

The use of SFI for the treatment of HF may be clinically effective and safe. However, this conclusion must be interpreted cautiously due to the generally low methodological quality and low evidence quality of the included SRs/MAs. More rigorously designed SRs/MAs and RCTs with high methodological quality are necessary for further proof.

## 1. Introduction

Heart failure (HF) is one of the leading causes of human morbidity and mortality worldwide. HF is a complex clinical syndrome with broad pathological processes, exhibiting an unpredictable trajectory and an escalating symptom profile along with time [1]. HF incidence remained stable in recent decades, with almost 26 million people suffered from heart failure around the world [2]. It was reported that approximately 10 per 1000 among those over 65 years of age in the United States [3] and 9 per 1000 of the population aged 35–74 years in China [4] have clinical manifestations HF.

Over the past 30 years, improvements in treatments that consist of some effective medicines, such as diuretics, digoxin, angiotensin-converting enzyme inhibitors (ACEIs), angiotensin receptor blockers (ARBs), and *β*-blockers, have improved survival and reduced the hospitalization rate in patients with HF. However, it cannot obtain a desired effect own to poor compliance, lower heart rate of patients, and other questions [5]. Considering the above multiple factors, a combination of Chinese herbal injection and western medicine (WM) treatment has already been a supportive measure in the treatment of HF in China. Shenfu Injection (SFI) has been used in treating cardiac diseases for a long time in China; pharmacological studies have suggested that SFI can reduce peripheral circulation resistance and improve microcirculation [6]. A literature search yielded several published systematic reviews (SRs)/meta-analyses (MAs) on SFI for HF, but their quality varied, and the evidence for the effectiveness of SFI is controversial. To comprehensively evaluate the evidence and applicability of the results of SRs/MAs on SFI for HF, we composed an overview.

## 2. Methods

### 2.1. Eligibility Criteria

#### 2.1.1. Type of Studies

All peer-reviewed, full-reported SRs/MAs based on randomized controlled trials were included. Duplicate reports, studies with the data were inconsistent or incomplete, and unavailable articles were excluded. No language limitation exists.

#### 2.1.2. Types of Participants

Participants with HF should be confirmed according to any internationally recognized or accepted clinical guidelines. There are no limitations in age, gender, race, or nationality.

#### 2.1.3. Types of Interventions

The intervention methods were SFI or SFI plus WM (e.g., cardiotonic, diuretic, ACEIs, *β*-blocker, and so forth); the control groups were treated with WM or blank controls.

#### 2.1.4. Types of Outcomes

SRs/MAs should have at least one clear outcome such as effective rate, left ventricular ejection fraction (LVEF) level, left ventricular diastolic diameter (LVDd) level, B-Natriuretic peptide (BNP) level, N-terminal pro-B-type nature tripeptide (NT-proBNP) level, 6-minute walk distance ((6-MWD), death, and adverse events.

### 2.2. Search Strategy

A systematic search was conducted in 8 databases including PubMed, Embase, the Cochrane Library, the web of science, China National Knowledge Infrastructure, Wanfang Database, Chongqing VIP, and Sino-Med from their establishment to May 17, 2020, with the following search terms: heart failure, shenfu injection, systematic review, and meta-analysis. We conducted an updated search on January 28, 2021, to provide more up-to-date and comprehensive evidence. Besides, we also search systematic review or meta-analysis registration website (https://www.crd.york.ac.uk/PROSPERO/) and checked the reference lists of all relevant SRs identified, and their authors were contacted to identify additional relevant SRs if necessary.  Table 1 provides a search strategy for the PubMed database.

### 2.3. Data Collection and Extraction

Two authors independently screened all potential abstracts and titles of reviews for inclusion, based on the selection criteria. Each review was evaluated independently, and the full texts of all potentially eligible were obtained for assessment to determine whether the review met the inclusion/exclusion criteria. Any disagreement regarding the possible inclusion/exclusion of any individual review was resolved by discussion with the third reviewer and by a final group consensus.

Two authors independently extracted data from eligible SRs/MAs. From each study, the following specific characteristics were extracted: the first author, year of publication, country, number of trials and participants and their characteristics, quality of the included trials (as reported by the review authors), interventions and comparisons relevant to this overview, outcomes relevant to this overview, quality assessment methods, and the summary estimate of the intervention effects. The corresponding authors were contacted by email for missing information.

### 2.4. Quality Assessment

Two authors separately evaluated the quality of included SRs/MAs by using Assessing the Methodological Quality of Systematic Reviews 2 (AMSTAR-2) [7]. AMSTAR-2 evaluates the systematic review using 16 distinct criteria, and seven of them are critical items. Each criterion of AMSTAR has 3 choices, namely, “yes,” “partial yes,” or “no.” When no or only 1 noncritical item did not conform, inferring rating overall confidence in the results of the review as high; when more than 1 noncritical item did not conform, inferring rating overall confidence in the results of the review as moderate; when 1 critical item did not conform with noncritical items conforming or not conforming, inferring rating overall confidence in the results of the review as low; and when more than 1 critical items did not conform with noncritical items conforming or not conforming, inferring rating overall confidence in the results of the review as critically low [7].

The evidence quality for each outcome measure was assessed with the Grade of Recommendation, Assessment, Development, and Evaluation (GRADE) [8] by two authors independently. Relevant evidence can be rated down for high risk of bias of included reviews, indirectness, imprecision, inconsistency, and publication bias. The GRADE assesses the certainty of the evidence for each outcome measures by categorizing evidence into “high,” “moderate,” “low,” or “very low” [9]. Any discrepancies were resolved by a final consensus among all reviewers. Descriptive analysis was used for efficacy evaluation.

## 3. Results

### 3.1. Study Selection

**Figure 1:**
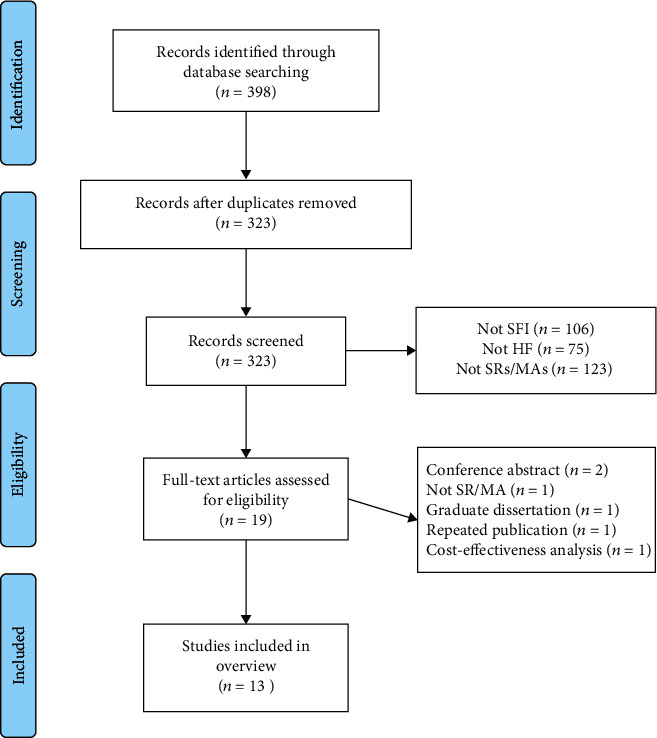
Flow diagram of the literature selection.

A total of 398 literatures were identified in initial search (Figure 1). After removing duplicates, there were 323 remained. By screened titles and abstracts, 304 literatures were excluded, and the remaining 19 literatures were eligible and then examined, respectively, among which 6 were further excluded, for the following reasons: 2 were conference abstract, 1 was a trail, 1 was a repeated publication, 1 was a graduate dissertation, and 1 was regarding to cost-effectiveness analysis. Finally, 13 SRs/MAs [10–22] were included in this overview.

### 3.2. Study Characteristics

The characteristics of included SRs/MAs are presented in Table 2. The included reviews were published between 2009 and 2020. Twelve SRs/MAs were published in Chinese [10–20, 22], and the remaining 1 [21] was in English. The number of RCTs included in the SRs/MAs varied widely, ranging from 8 to 97 studies, and the sample size varied from 559 to 8272 participants. The intervention of the control groups was WM treatment, for instance, ACEIs, *β*-blocker, cardiotonic, and diuretic. In the meantime, the treatment groups received SFI on the basis of the control groups. The methodological quality of the original studies using various appraisal tools was assessed in all SRs/MAs as mainly fair or poor.

### 3.3. Methodological Appraisal

An overview of methodological quality of included SRs/MAs is given in Table 3. All SRs/MAs were regarded as critically low quality. The result of AMSTAR-2 showed that the key factors affecting the quality of the reviews included were item 2 (none review contained an explicit statement that the review methods were established prior to the conduction of the review and justified any significant deviations from the protocol), item 4 (only 3 included studies provide the use of a specific search strategy), item 7 (all review authors did not provide a list of excluded studies and justified the exclusions), and item 15 (two reviews did not consider the publication bias when the authors interpreted or discussed the study results).

### 3.4. GRADE Evidence Quality Classification

The quality of evidence for 46 outcomes in 13 included SRs/MAs is presented in Table 4. Of these outcomes, the quality of evidence was high in 0 (0/51, 0%), moderate in 27 (27/51, 52.9%), low in 18 (18/51, 35.3%), and very low in 6 (6/51, 11.8%). The evidence level of all concerned outcomes was downgraded due to the study limitations within the original trials, inconsistency, imprecision, and the possibility of publication bias. Details regarding downgrades for each GRADE domain by outcome are given in Table 4.

### 3.5. Description of Efficacy

#### 3.5.1. Effectiveness of SFI for HF

We summarized the outcomes from the included SRs/MAs and presented them in Table 4. The evidence in eleven SRs/MAs [11–22] suggested that the effective rate of SFI plus WM was superior to WM alone. Eleven SRs/MAs [10–12, 14–18, 20–22] reported the outcomes for LVEF; meta-analysis showed that the SFI group was better than control group in increasing LVEF. Similarly, 6 SRs/MAs [10, 12, 14–17] reported the outcomes for LVDd; results showed that the SFI group was better than the control group in increasing LVDd. For BNP level, it was reported in 7 SRs/MAs [10–12, 14, 15, 18, 22] that BNP levels of the SFI group were significantly lower than the control group. Similarly, 3 SRs/MAs [12, 15, 22] reported that NT-proBNP levels of the SFI group were significantly lower than the control group. Four SRs/MAs [13, 15–17] assessed 6-MWD of patients who received SFI or WM treatment; consistent results showed significant increase in walking distance in the SFI group. Night reviews [12–14, 17–22] compared the effects of SFI plus WM versus WM alone using the TCM symptom score; the results showed that the combined treatment had a greater effect than CM alone. One review [13] revealed that there was a significantly greater reduction in MLHFQ score in the SFI group than in the control group. Readmission rates were reported in 1 review [13]; meta-analysis showed that there was statistical significance between the SFI group and the control group. Mortality rate was reported in 2 reviews [13, 21]. Song et al. [21] found that SFI can significantly reduce mortality of patients; however, another review [13] reported no significant difference between the SFI group and the control group.

#### 3.5.2. Safety of SFI for HF

Of all included SRs/MAs, 8 reviews [10, 11, 16–18, 20–22] mentioned the adverse events of SFI for HF. Qualitative descriptive analysis was performed due to the small number of studies. Five SRs/MAs reported no adverse events were found in the SFI group. However, the remaining 3 SRs/MAs [11, 20, 21] reported the following symptoms of side effects including dry mouth, dryness heat, fullness of the head, insomnia, dysphoria, skin itching, tachycardia, feverish dysphoria, flushing of face, tidal fever, dizziness due to low blood pressure, gastrointestinal discomfort, and palpitation.

## 4. Discussion

The impairment of HF has been a global public health issue; with the utilization of conjunction between SFI and WM in its treatment, the efficacy of HF has been promoted; meanwhile, more and more relevant SRs/MAs were carried out. Thus, the vast number of SRs/MAs on this topic is concerning, particularly those of low quality which may propagate inaccurate or biased results and conclusions. Under the circumstances, this approach of synthesizing findings of SRs/MAs is better than a high number of SRs/MAs with low quality and unconvincing conclusions, thereby providing a comprehensive evidence-based summary on evident outcomes. In addition, an overview may provide notable information to guide future high-quality RCTs or SRs/MAs.

### 4.1. Major Study Findings

This is the first overview of SRs/MAs that investigate the effectiveness and safety of SFI for HF. We rigorously appraised the published SRs/MAs with AMSTAR-2 and GRADE. For AMSTAR-2, all included SRs/MAs were judged to be of critically low quality. The key factors affecting the quality of the reviews included were item 2, item 4, item 7, and item 15. Based on GRADE, the quality of evidence was high in 0 (0/51, 0%), moderate in 27 (27/51, 52.9%), low in 18 (18/51, 35.3%), and very low in 6 (6/51, 11.8%). The evidence level of all concerned outcomes was downgraded. The study limitations within the original trials was the most common of the downgrading factors of the evidence level, followed by inconsistency, the possibility of publication bias, and imprecision. Descriptive analysis showed that SFI was an effective and safe method for HF.

### 4.2. Implications for Clinical Practice

This overview included 51 outcome measures and almost all of which reached positive conclusions; all included SRs/MAs indicated that SFI is effective in treating HF. However, the evidence level of all concerned outcomes was unsatisfactory, indicating that the conclusions of included SRs/MAs may differ from the true results; the lower the quality is the more likely further research would change our confidence in the estimates and the estimates themselves [23].

Additionally, AMSTAR-2 results showed that the methodology quality of all included SRs/MAs was critically low. As we know, high quality of SRs/MAs is crucial to ensure validity, clarity, and accurate comprehension of evidence, while low-quality SRs/MAs are the opposite. Furthermore, authors of most SRs/MAs did not wish to draw definitive conclusions due to the small size of the included trials or their low quality. Therefore, definitive conclusions were impossible to draw from published results; caution should be warranted when recommending SFI as an alternative treatment for HF.

### 4.3. Implications for Further Study

Based on AMSTAR-2, the key factors affecting the quality of the reviews included were item 2 (none review contained an explicit statement that the review methods were established prior to the conduction of the review and justified any significant deviations from the protocol). The previous study [24] has shown that research protocols help to increase the transparency of the study methods and improve the overall methodological quality of SRs/MAs. Item 4 (only 3 included studies provide the use of a specific search strategy) is likely contributed to generating publication bias and undermined the conclusion's reliability. Item 7 (all review authors did not provide a list of excluded studies and justified the exclusions) is likely contributed to leaving some information missing and undermined the conclusion's reliability. Item 15 (2 reviews did not consider the publication bias when the authors interpreted or discussed the study results) may affect the credibility of the final results. Thus, future SRs/MAs should address these identified shortcomings. Researchers should ensure that the AMSTAR-2 is strictly followed before publication.

Based on GRADE, the evidence level of all concerned outcomes was downgraded. The limitations within the original trials were the most common of the downgrading factors. Although all the interventions were from RCTs, results showed that there is much room for addressing the bias in random, distributive hiding, or blind during the RCT process; well-designed and implemented RCTs are considered gold standards for evaluating interventions to minimize or avoid bias [25]. Additionally, authors of most SRs/MAs declared that more high-quality RCTs with large-sample size should be carried out.

### 4.4. Strength and Limitations

As the highest source of evidence, this overview will be beneficial to clinicians in making decisions in opting for methods treating the disease and help researchers to improve the quality of their study. Widely used validated tools to assess methodology (AMSTAR-2) and quality of evidence (GRADE) of included reviews were used; however, valuation of methodological quality and quality of evidence was a subjective process; the accuracy of assessor's assessments cannot be guaranteed.

## 5. Conclusion

The use of SFI for the treatment of HF may be clinically effective and safe. However, this conclusion must be interpreted cautiously due to the generally low methodological quality and low evidence quality of the included SRs/MAs. More rigorously designed SRs/MAs and RCTs with high methodological quality are necessary for further proof.

## Figures and Tables

**Table 1:** Search strategy for the PubMed database.

Query	Search term
# 1	Heart failure [Mesh]
# 2	Heart failure[Title/Abstract] OR cardiac failure[Title/Abstract] OR decompensation heart[Title/Abstract] OR myocardial failure [Title/Abstract] OR dyspnea, paroxysmal[Title/Abstract] OR edema, cardiac[Title/Abstract] OR left sided heart failure[Title/Abstract] OR right sided heart failure[Title/Abstract]
# 3	#1 OR #2
# 4	Shenfu injection[Title/Abstract] OR shenfu[Title/Abstract]
# 5	Meta-analysis as Topic[Mesh]
# 6	Systematic review[Title/Abstract] OR meta-analysis[Title/Abstract] OR meta analysis[Title/Abstract] OR meta-analyses
# 7	#5 OR #6
# 8	#3 AND #4 AND #7

**Table 2:** Characteristics of the included reviews.

Author, year	Country	Trials (sample size)	Treatment intervention	Control intervention	Quality assessment tool	Conclusion summary
Wu [10] 2018	China	10 (851)	SFI + WM	WM	Cochrane criteria	The combined treatment of SFI and WM can significantly improve TCM syndrome, reduce the BNP level, improve the LVEF level, and improve the hemodynamic indicator among patients with HF. However, firm conclusion towards the validity and safety of SFI cannot be drown owing to the low quality of included trails.
Jia et al. [11] 2018	China	17 (1286)	SFI + WM	WM	Jadad	On the basis of routine treatment of WM, SFI is more effective than WM alone in the treatment of acute left HF.
Wen et al. [12] 2017	China	21 (1630)	SFI + WM	WM	Jadad	The efficacy of routine treatment of WM combined with SFI in the treatment of HF is better than that with WM alone.
Ma et al. [13] 2017	China	19 (1829)	SFI + WM	WM	Jadad	SFI is unable to reduce the mortality of chronic HF, but it can significantly improve the quality of life.
Luo et al. [14] 2015	China	25 (1975)	SFI + WM	WM	Jadad	The curative effect of the treatment on patients with HF with WM plus SFI is better than WM alone.
Du and Dai [15] 2014	China	24 (1743)	SFI + WM	WM	Jadad	SFI can significantly improve the clinical efficacy, but which needs to be further confirmed by more large-sample, high-quality RCTs.
Xu et al. [16] 2013	China	8 (559)	SFI + WM	WM	Cochrane criteria	The combination of SFI and WM can highly improve the efficacy of HF in old patients.
Huang [17] 2011	China	28 (2070)	SFI + WM	WM	Jadad	SFI can increase the treatment effective rate of HF and improve heart function. However, this conclusion is limited owing to the poor quality of the included studies.
Hou et al. [18] 2011	China	16 (1117)	SFI + WM	WM	Cochrane criteria	The therapeutic effect of combining WM with SFI on HF patients is better than that of WM alone.
Bin [19] 2010	China	8 (875)	SFI + WM	WM	Jadad	Compared with WM, the combined treatment of SFI and WM is more effective in the treatment of HF and can significantly improve the clinical symptoms.
Ma et al. [20] 2009	China	70 (5294)	SFI + WM	WM	Jadad	SFI is one of the important and effective drugs for the treatment of cardiac insufficiency, and the conclusion is reliable.
Song et al. [21] 2012	China	97 (8272)	SFI + WM	WM	Cochrane criteria	SFI appears to be effective for treating HF. However, further rigorously designed RCTs are warranted because of insufficient methodological rigor in the majority of included trials.
Guo et al. [22] 2020	China	22 (1753)	SFI + WM	WM	Cochrane criteria	SFI combined with WM can improve the clinical efficiency, reduce BNP, NT-proBNP levels, and improve cardiac function with good safety. Due to limited quality and quantity of the included studies, more studies are required to verify the conclusions above.

**Table 3:** Result of the AMSTAR-2 assessments.

Author, year	AMSTAR-2																Quality
	Q1	Q2	Q3	Q4	Q5	Q6	Q7	Q8	Q9	Q10	Q11	Q12	Q13	Q14	Q15	Q16	
Wu [10] 2018	Y	PY	Y	PY	Y	Y	N	Y	Y	N	Y	Y	Y	Y	N	N	CL
Jia et al. [11] 2018	Y	PY	Y	Y	Y	Y	N	Y	Y	Y	Y	Y	Y	Y	Y	Y	CL
Wen et al. [12] 2017	Y	PY	Y	PY	Y	Y	N	Y	Y	Y	Y	Y	Y	Y	Y	Y	CL
Ma et al. [13] 2017	Y	PY	Y	PY	Y	Y	N	Y	Y	N	Y	Y	Y	Y	Y	N	CL
Luo et al. [14] 2015	Y	PY	Y	PY	Y	Y	N	Y	Y	Y	Y	Y	Y	Y	Y	Y	CL
Du and Dai [15] 2014	Y	PY	Y	PY	Y	Y	N	Y	Y	N	Y	Y	Y	Y	Y	Y	CL
Xu et al. [16] 2013	Y	PY	Y	PY	Y	Y	N	Y	Y	N	Y	Y	Y	Y	N	N	CL
Huang and Xu [17] 2011	Y	PY	Y	PY	Y	Y	N	Y	Y	N	Y	Y	Y	Y	Y	N	CL
Hou et al. [18] 2011	Y	PY	Y	Y	Y	Y	N	Y	Y	Y	Y	Y	Y	Y	Y	Y	CL
Bin [19] 2010	Y	PY	Y	PY	Y	Y	N	Y	Y	N	Y	Y	Y	Y	Y	N	CL
Ma et al. [20] 2009	Y	PY	Y	PY	Y	Y	N	Y	Y	N	Y	Y	Y	Y	Y	N	CL
Song et al. [21] 2012	Y	PY	Y	PY	Y	Y	N	Y	Y	N	Y	Y	Y	Y	Y	PY	CL
Guo et al. [22] 2020	Y	PY	Y	Y	Y	Y	N	Y	Y	Y	Y	Y	Y	Y	Y	Y	CL

**Table 4:** Certainty of evidence of SRs/MAs included.

Author; year	Outcomes	Limitations	Inconsistency	Indirectness	Imprecision	Publication bias	Relative effect (95% CI)	Quality
Wu [10] 2018	TCM symptom score	-1^①^	0	0	0	0	OR 3.79 (2.19, 6.57)	M
	BNP level	-1^①^	-1^②^	0	0	0	OR -180.16 (-257.41, -102.91)	L
	LVEF	-1^①^	0	0	0	0	OR 5.53 (3.99, 7.07)	M
	LVDd	-1^①^	0	0	-1^③^	-1^④^	OR -1.73 (-3.54, 0.07)	CL

Jia et al. [11] 2018	Effective rate	-1^①^	0	0	0	-1^⑤^	RR 1.26 (1.14, 1.38)	M
	LVEF	-1^①^	0	0	0	-1^⑤^	WMD 7.18 (4.70, 9.66)	L
	BNP level	-1^①^	-1^②^	0	0	0	WMD = 125.62, 95% CI 75.86, 175.37	L

Wen et al. [12] 2017	Effective rate	-1^①^	0	0	0	-1^⑤^	OR 1.22 (1.16, 1.27)	L
	TCM symptom score	-1^①^	0	0	0	-1^⑤^	OR 2.94 (1.71, 5.04)	L
	LVEF	-1^①^	-1^②^	0	0	0	WMD 4.12 (3.00, 5.24)	L
	LVDd	-1^①^	-1^②^	0	-1^③^	-1^④^	WMD -2.50 (-4.57, -0.43)	CL
	BNP level	-1^①^	-1^②^	0	0	0	WMD -108.73 (-145.93, -71.52)	L
	NT-proBNP level	-1^①^	-1^②^	0	0	0	WMD -121.52 (-180.61, -62.40)	L

Ma et al. [13] 2017	Effective rate	-1^①^	0	0	0	0	OR 3.38 (2.47, 4.61)	M
	MLHFQ score	-1^①^	-1^②^	0	0	0	MD -5.57 (-8.26, -2.87)	L
	6-MWT	-1^①^	0	0	0	0	MD 44.65 (41.27, 48.03)	M
	Readmission rates	-1^①^	0	0	0	0	OR 0.42 (0.29, 0.59)	M
	Mortality rate	-1^①^	0	0	0	0	OR 0.59 (0.31, 1.13)	M

Luo et al. [14] 2015	Effective rate	-1^①^	0	0	0	-1^⑤^	OR 3.55 (2.69, 4.69)	L
	LVEF	-1^①^	-1^②^	0	0	0	WMD 5.78 (3.86, 7.70)	L
	LVDd	-1^①^	0	0	0	0	WMD -1.52 (-2.43, -0.61)	M
	BNP level	-1^①^	-1^②^	0	0	0	WMD -98.30 (-143.81, -52.78)	L

Du and Dai [15] 2014	Effective rate	-1^①^	0	0	0	-1^⑤^	RR 1.26 (1.20, 1.32)	L
	LVEF	-1^①^	0	0	0	0	WMD 3.67 (3.31, 4.21)	M
	LVDd	-1^①^	0	0	0	0	WMD -2.03 (-2.76, -1.31)	M
	BNP level	-1^①^	0	0	0	0	WMD -94.20 (-101.43, -86.97)	M
	NT-proBNP level	-1^①^	0	0	0	0	WMD -317.75 (-347.06, -288.44)	M
	6-MWT	-1^①^	0	0	-1^③^	-1^④^	WMD 47.32 (29.11, 65.53)	CL

Xu et al. [16] 2013	Effective rate	-1^①^	0	0	0	0	RR 1.20 (1.11, 1.29)	M
	LVEF	-1^①^	-1^②^	0	0	0	MD 4.79 (-0.07, 9.65)	L
	LVDd	-1^①^	0	0	0	0	MD 5.90 (3.97, 7.84)	M
	6-MWT	-1^①^	-1^②^	0	0	0	MD 62.48 (43.12, 81.84)	L

Huang and Xu [17] 2011	Effective rate	-1^①^	0	0	0	0	OR 3.10 (2.42, 3.98)	M
	LVEF	-1^①^	0	0	0	0	WMD 5.80 (3.28, 8.33)	M
	LVDd	-1^①^	0	0	0	0	WMD -1.66 (-3.01, -0.31)	M
	6-MWT	-1^①^	0	0	0	0	WMD 21.26 (7.64, 34.88)	M

Hou et al. [18] 2011	Effective rate	-1^①^	0	0	0	0	RR 3.30 (2.22, 4.92)	M
	TCM symptom score	-1^①^	0	0	-1^③^	-1^④^	RR 6.85 (2.90, 16.17)	CL
	LVEF	-1^①^	0	0	0	0	WMD 3.54 (2.78, 4.30)	M
	BNP level	-1^①^	0	0	0	0	WMD 34.69 (1.78, 67.60)	M

Bin [19] 2010	Effective rate	-1^①^	0	0	0	-1^⑤^	OR 2.41 (1.66, 3.48)	L

Ma et al. [20] 2009	Effective rate	-1^①^	0	0	0	0	OR 3.19 (2.71, 3.7)	M
	LVEF	-1^①^	0	0	0	0	OR 5.61 (3.69, 8.52)	M

Song et al. [21] 2012	Effective rate	-1^①^	0	0	0	0	RR 1.19 (1.17, 1.21)	M
	Mortality rate	-1^①^	0	0	0	0	RR 0.52 (0.37, 0.74)	M
	LVEF	-1^①^	0	0	0	0	WMD 6.31 (5.18, 7.44)	M

Guo et al. [22] 2020	Effective rate	-1^①^	0	0	0	0	RR 1.22 (1.15, 1.28)	M
	BNP level	-1^①^	-1^②^	0	0	0	MD -139.923 (-186.89, -92.96)	L
	NT-proBNP level	-1^①^	-1^②^	0	0	0	MD -442.41 (-601.95, -282.88)	L
	LVEF	-1^①^	-1^②^	0	0	-1^④^	MD 4.22 (3.67, 4.78)	CL
	TCM symptom score	-1^①^	0	0	-1^③^	-1^④^	MD -2.11 (-2.93, -1.29)	CL

LVEF: left ventricular ejection fraction; LVDd: left ventricular diastolic diameter; BNP: B-Natriuretic peptide; NT-proBNP: N-terminal pro-B-type nature tripeptide, 6-MWD: 6-minute walk distance; TCM: Traditional Chinese Medicine; MLHFQ: Minnesota Living with Heart Failure Questionnaire; RR: risk ratio; OR: odds ratio; SMD: standardized mean difference; WMD: weighted mean difference; MD: mean difference; CL: critical low; L: low; M: moderate; H: high. ^①^The design of the experiment with a large bias in random, distributive hiding, or blind. ^②^The confidence interval overlaps less, the heterogeneity test *P* is very small, and the *I*^2^ is larger. ^③^Confidence interval is not narrow enough, or the sample size is small. ^④^Fewer studies are included, and there may be greater publication bias. ^⑤^Funnel graph asymmetry.

## Data Availability

All data generated or analyzed during this study are included in this article. No funding was received for this research.

## Abbreviations

SFI: Shenfu Injection
HF: Heart failure
SR: Systematic review
MA: Meta-analysis
AMSTAR-2: Assessing the Methodological Quality of Systematic Reviews 2
GRADE: Grading of Recommendations, Assessment, Development, and Evaluation
RCTs: Randomized clinical trials
ACEIs: Angiotensin-converting enzyme inhibitors
ARBs: Angiotensin receptor blockers
WM: Western medicine
LVEF: Left ventricular ejection fraction
LVDd: Left ventricular diastolic diameter
BNP: B-Natriuretic peptide
NT-proBNP: N-Terminal pro-B-type nature tripeptide
6-MWD: 6-minute walk distance
TCM: Traditional Chinese Medicine
MLHFQ: Minnesota Living with Heart Failure Questionnaire.
